# Remarks on Proposed Amendments in the Lunacy Act Relating to Single Patients

**Published:** 1903-01-24

**Authors:** F. St. John Bullen


### HOSPITAL CLINICS AND MEDICAL PROGRESS.

### REMARKS ON PROPOSED AMENDMENTS IN THE LUNACY ACT RELATING TO SINGLE PATIENTS.

By F. ST. JOHN BULLEN, M.R.C.S.

THE recent address given by Sir W. Gowers on "Lunacy and the Law" and its subsequent discussion at the meeting of the Medico-Psychological Society, manifested afresh the occasional injustice suffered under the existing law by persons whose mental state may only need them to be subjected to a modified supervision and treatment. This is only obtainable by conforming to the same procedures and submitting to the same expense and civil disqualification as is involved in pronounced insanity. The law forbids a person to take charge of anyone not under certificates but certifiably insane, if they receive payment for the same, and this wise provision has extinguished countless abuses. There are, or may be, however, two undesirable results: First, that certain persons who would be better under control are not so by reason of difficulty or inadvisability of certification; and secondly, that some may be placed under certificates unnecessarily. There is no middle course between allowing the patient full liberty, and legally depriving him of it entirely. For evidence (under the Statute though not under Common Law) of mental unsoundness justifying the detention under care and treatment is enough, apart from proof of directly prejudicial conduct; and there may be cases where at the request of the relatives, the medical man may feel himself acting conscientiously in seeking anxiously for a sufficiency of testimony to fulfil requirements. Nor can there be any doubt, as Sir W. Gowers has stated, that the procedure involved in certification and the knowledge which cannot be hidden from many patients, that this has constituted them in the eye of the law as being of unsound mind and liable to detention in an asylum or elsewhere, and has deprived them of civil abilities, must influence their condition very prejudicially. Another disadvantage which attends the present system of enforced certification of even mild degrees of unsoundness of mind is the expense incurred. This must needs be considerable to make a recompense where a good deal of trouble and responsibility is incurred by the person in charge in carrying out the provisions of the Act. This must debar many suitable cases from the advantages of private care and treatment. Dr. Gowers indicates two lines of procedure which he thinks might be adopted in the case of persons not sufficiently insane to need certification with its attendant limitations. These are the substitution of a system of notification, or the retention of the present forms of procedure, with a simple alteration in the certificate which should delete the authority to detain. The former process is in use in Scotland, where a notification by a medical practitioner of an unsound mental condition (likely to be improvable) of the person received for treatment in private care is a sufficient warrant, if satisfactory to the Lunacy Board, for six months. The statement to be satisfactory must be to the effect that the patient's malady is not confirmed and does not require the use of coercive measures. Cases of confirmed disorder are not allowed to be received under this notification system.

This plan appears to work well in Scotland, where the boarding-out system is in vogue, and this is perhaps the best answer to any objections raised against it. It may, however, be well to consider the possible disadvantages that might arise without the provision of safeguards, in view of the much larger numbers who might take advantage of such a system in England and the consequent difficulty in watching its satisfactory operation. It would seem proper that with the notification system a considerable amount of supervision by the Lunacy Board or its representatives should still be exercised. In the first place, as cases of incipient insanity would be especially likely to form a large proportion of those coming under its provision, it would be essential to secure for their proper treatment evidence that adequate control and supervision were used. For it must be remembered that under the simple conditions of this system numbers of persons would probably cater and perhaps insufficiently, for such of this class of cases who could afford but small payments. With the present demands of the law, the charges usually requested for the private care of a certified patient are considerable enough to make reasonable comfort and skilled attention almost a matter of course. In the second place periodical medical reports would be needful, though perhaps less often than in certified cases, in order that no attempt should be made to harbour a patient who had become either more, or less, unsound than contemplated by the system. It will be prevised that neither the system adopted in Scotland, nor the certificate of insanity without power of detention is a satisfactory settlement of the question. For many mild cases otherwise suitable as regards the first method of procedure are chronic and therefore inadmissible. And as regards the second the query arises, if the patient is certified unsound of mind and thus deprived of civil responsibility, how are his actions to be controlled in default of the power to detain (in its broad or narrow sense), and again who will be bold enough, amongst general practitioners at any rate, to certify a patient insane and yet not needing authoritative control? Moreover, the stigma of insanity is little if at all lessened by this modification.

(*To be continued.*)

